# Mental health prevention program for Roma children and their families fleeing from Ukraine

**DOI:** 10.1192/j.eurpsy.2023.148

**Published:** 2023-07-19

**Authors:** J. Balazs, Nóra Kollárovics, Bernadett Frida Farkas, Xénia Roszik-Volovik, Eszter Frank-Bozóki

**Affiliations:** Institute of Psychology, Eotvos Lorand University, Budapest, Hungary

## Abstract

**Abstract:**

The Childhood Mental Disorders Research Group of ELTE began helping displaced children and their families immediately after the outbreak of war in Ukraine in March 2022. This work is still being carried out on a voluntary basis. According to the current needs, they organize "playgroup" for children and their families several times a week both at a fixed location at the ELTE and at external locations in shelters where they work with many children and their families at the same time. The main focus of the groups is the prevention of mental illness.

In this presentation, the work of the Research group will be presented, which has been done in a shelter that accommodates mainly families with Roma ethnic backgrounds from the Transcarpathia region fleeing the Russian-Ukraine war.

**Disclosure of Interest:**

None Declared

